# The circadian rhythm of heart rate variability in academic teachers with anxiety disorders

**DOI:** 10.13075/ijomeh.1896.02689

**Published:** 2026

**Authors:** Anna Janocha, Robert Skalik, Aldona Molęda, Aleksander Stal, Dariusz Kałka

**Affiliations:** 1 Wrocław University of Science and Technology, Department of Preclinical Sciences, Pharmacology and Medical Diagnostics, Faculty of Medicine, Wrocław, Poland; 2 Wroclaw Medical University, Clinical Department of General Surgery, Faculty of Medicine, Wrocław, Poland; 3 Wroclaw University of Health and Sport Sciences, Department of Physiotherapy in Internal Medicine and Oncology, Faculty of Physiotherapy, Wrocław, Poland

**Keywords:** panic disorder, generalized anxiety disorder, academic teachers, 24-hour Holter analysis, circadian rhythm of heart rate variability, markers of autonomic functions

## Abstract

**Objectives:**

The academic teachers are increasingly diagnosed with anxiety disorders, which results from a high level of work-related stress. These disorders can be both an independent risk factor of many somatic conditions and the cause of complications of various organic disorders leading to poor prognosis. The purpose of this study is to compare the heart rate variability (HRV) recorded both during the day and at night in the academic teachers with anxiety disorders and healthy individuals.

**Material and Methods:**

The study was conducted on 38 academic teachers – consecutive outpatients with anxiety disorders who participated in the intensive group psychotherapy. The diagnosis was made according to *Diagnostic and statistical manual of mental disorders, fourth edition, text revision* (DSM-IV-TR) criteria using the *Present State Examination* (PSE-10) questionnaire. Seventeen examined patients were diagnosed with panic disorder (PD) and 21 with generalized anxiety disorder (GAD). The control group consisted of 40 healthy academic teachers. The resulting 3 groups were compared in terms of circadian HRV using the Oxford Medilog Suprima Holter System.

**Results:**

The HRV analysis showed the nocturnal falls in the time and frequency parameters related to the parasympathetic activity, e.g., high frequency. The most unfavorable alterations were observed in the PD group, which comprised the disappearance of day vs. night amplitude of the majority of HRV parameters. Hence, the PD group was particularly susceptible to the arrhythmic events. It was noticeable that nocturnal vagotonia in the PD group was significantly lower as compared with the GAD and control group.

**Conclusions:**

The innovative aspect of the present study was to find a distinct profile of HRV with regard to the types of anxiety disorders because of the existing considerable differences between the nature of PD and GAD.

## Highlights

- Occupational stress among academic teachers affects their personal health.
- The influence of work-related stress on the presence of anxiety disorder was noted.
- Diurnal heart rate variability (HRV) was flattened in patients with anxiety disorders.
- In the panic patients a lack of diurnal rhythm of the autonomic regulation was noted.
- A different profiles of HRV with regard to the types of anxiety disorders were found.

## INTRODUCTION

The modern civilization contributes to the development of a long-term and nearly permanent social stress that can be an independent risk factor for poor physical and mental well-being. Work-related stress often affects the academic teachers and make professional work difficult or impossible. Stress results from didactic duties, as well as strong competition in the scientific research and formal requirements of the university [1]. In general, occupational stress indicates a harmful long-lasting imbalance between the individual's resources such as knowledge, abilities or skills, and the work demands.

There are many occupational stress models and theories. The Siegrist's effort-reward imbalance (ERI) and Karasek's high demand – low control theory are the most popular concepts among them. In Karasek's model, a workplace stress results from the combination of high requirements of a given person's job and low control over his or her own responsibilities. The ERI model states that work-related stress depends upon the interrelationship between efforts and rewards at work – hard work without adequate appreciation causes a sense of harm and stressful imbalance [2]. In turn, according to Beck's cognitive theory of psychopathology, thoughts of failure and cognition of harm are unique predictors of anxiety. The course of anxiety disorders points at the importance of environmental influences (e.g., occupational stress) as triggering factors, and cognitive and psychophysiological mechanisms of the symptoms perpetuation [3].

In terms of psychophysiology, a stressful situation triggers the release of stress hormones and overactivity of sympathetic autonomic nervous system (ANS). The ANS does not have patterns that correspond with specific emotions, although the variability in the parasympathetic and sympathetic activation across the instances of emotions is the norm [4]. Mental stress has been found related to the increased low frequency (LF) heart rate variability (HRV) power reflecting the increase in the sympathetic activity. Hence, changes related to the psychophysiological strain of the ANS can be evaluated by HRV analysis [2].

Mental stress and burnout, leading to anxiety and depression, are perhaps the best established triggers of psychogenic cardiovascular conditions [1]. The acute mental stress can cause a unique form of a transient cardiomyopathy known as Takotsubo cardiomyopathy or “broken heart syndrome” – a transient left ventricular dysfunction [5]. Furthermore, the cardiovascular consequences of anxiety disorders including panic disorder (PD) and generalized anxiety disorder (GAD) are also well known. In academic teachers with anxiety disorders, recurrent cardiac events are common and associated with not only worse health-related quality of life, but also the unfavorable course of the primary disease and the worsening prognosis that may lead to higher mortality [1, 6].

The activation of various neurogenic pathways is an important mediator of the acute and chronic stress-induced heart diseases [6, 7]. The dysfunction of ANS is predominantly implicated in the pathophysiology of the 2 afore-mentioned diseases. Autonomic nervous system plays an es-sential role in the regulation of the circulatory system. The increased sympathetic activation is the most common cause of the stress-induced cardiac arrhythmias. The anxiety disorders, especially PD, have been shown to be directly linked to the development of the cardiac arrhythmias [8]. On the other hand, the anxiety disorders can be complicated by autonomic disorders with the elevated sympathetic control as a result of decreased vagal tone [6, 7, 9]. Such imbalance within the ANS has a significant impact on the HRV – the physiological phenomenon of variation of the heart beats.

The psychiatric disorders (including PD and GAD) are associated with a whole array of impairments including dysregulation of the autonomic or involuntary functions such as heart rate, blood pressure and respiration. The incidence rates of PD and GAD have been increasing each year. However, to date, these disorders have been diagnosed only by symptoms and no objective biomarkers have been used to distinguish between them. The HRV is currently being investigated as a candidate biomarker of various anxiety disorders. Although originally HRV was used to assess the severity of cardiovascular disease and then extended to the field of mental illness. Nowadays, the HRV is one of the most promising markers of autonomic function. It is thought that HRV may serve as a surrogate measure of balance between the brain and the cardiovascular system [10–12].

Many authors suggest that there is an autonomic dysfunction with the characteristic changes of HRV profile in patients with PD and GAD. In the course of both mentioned diseases a decrease of total HRV and its components reflecting parasympathetic activity root mean square of successive RR interval differences (rMSSD), percentage of RR intervals that differ by >50 ms (pNN_50_) and high frequency (HF) were observed. These trends indicate that low vagal activity is a robust feature of anxiety disorders. Some studies have revealed that psychological stimuli could amplify the above HRV changes in psychiatric patients. In other words, these patients' low vagal activity may result from their psychological distress. A reduction of the vagal impact on the cardiovascular regulation is prognostically unfavorable [9, 11, 12]. Some authors also reported decreased very low frequency (VLF) and LF power in PD patients as compared with controls over the 24-hour period. The significantly lower mean (M) of the standard deviation (SD) of all the NN intervals for each 5 min (SDNN-i), which correlates with the VLF power, was also observed. Meanwhile, the SD of NN (SDNN) intervals was significantly lower in these patients during sleep [13, 14]. Sloan et al. [15] reported that the decrease in nocturnal HRV measures was more marked in anxiety disorder patients as a whole than in the control subjects. The results of other studies showed that PD and GAD patients exhibited significantly reduced HRV as compared with controls. The research also revealed significant differences in HRV indices (rMSSD, SDNN and the LF/HF ratio); the authors demonstrated that PD and GAD patients had a significantly lower SDNN and rMSSD, meanwhile, the LF/HF ratio was significantly higher as compared with controls. Another finding of their analysis was that GAD patients had significantly lower SDNN than PD patients. Many studies have shown a reduced HRV in patients with GAD and PD due to the elevated sympathetic control and reduced vagal control [10–12]. Heart rate variability monitoring provides a useful tool for measuring autonomic functions also in the occupational stress context [16].

The aim of the present study was to assess the functioning of the ANS, including circadian rhythm disturbances in people with PD and GAD and also evaluate physiological correlates of anxiety disorders with regard to the disturbances of autonomic regulation of cardiovascular function. At first, the ANS activity profile was assessed in the PD and GAD patient groups using HRV indices. Followingly, an attempt was made to assess the circadian rhythm of HRV profile as a biomarker of autonomic function in different types of anxiety disorders. The obtained results were compared with the circadian rhythm of HRV in the controls. In order to obtain the optimal intergroup assessment, the verification process using the objective methods such as 24-hour continuous electrocardiogram (ECG) Holter monitoring with evaluation of HRV was performed.

## MATERIAL AND METHODS

### Study group

Thirty eight academic teachers – consecutive outpatients suffering from anxiety disorders participating in the intensive group psychotherapy in the day ward of the Lower Silesian Center for Mental Health in Wrocław, Poland, entered the study. The patients diagnosed with anxiety disorders were referred to the psychotherapy day ward by psychiatrists, mainly due to the inefficacy of pharmacological treatment and individual psychotherapy. The short-term psychodynamic group psychotherapy with 2 sessions every working day lasted for 12 weeks.

The provisional psychiatric diagnosis was made on the basis of long-term observation lasting at least 3 months, and then verified at the beginning of therapy after admission to the day ward during 4-week observation period. The final psychiatric diagnoses were made using the *Present State Examination* (PSE-10) questionnaire with application of *Diagnostic and statistical manual of mental disorders, fourth edition, text revision* (DSM-IV-TR) criteria and then verified by a psychotherapeutic team at the end of treatment. The PSE-10 questionnaire is a part of the *Schedules for Clinical Assessment in Neuropsychiatry* (SCAN) aimed at assessing, measuring and classifying psychopathology and behavior associated with major psychiatric disorders in the adults [17]. The Polish adaptation of SCAN was a collateral result of the international European Day Hospital Evaluation (EDEN) project and as such was successfully tested in the clinical setting [18, 19]. The PSE-10 served as a diagnostic tool for PD and GAD according to the DSM-IV-TR criteria regarding the time of 24-hour continuous ECG recordings. Seventeen patients with PSE – based diagnosis of PD and 21 patients of GAD were enrolled consecutively in the middle of psychotherapeutic process (between weeks 5 and 8).

Forty academic teachers in the control group, with a negative history of anxiety disorders, were selected on the occasion of research conducted in the Department of Pathophysiology of Wroclaw Medical University, Wrocław, Poland, and subjected to the same tests as patients with anxiety disorders. The significant differences between the examined groups in terms of sex, age and BMI were not found.

None of the people included in any of the groups had a diagnosis of arterial hypertension or cardiovascular disease. Patients with a history of significant atrial or ventricular arrhythmia, other heart diseases, kidney disease, diabetes mellitus, sleep apnea syndrome, any neurological disease, psychotic disorders or a history of psychotic disorders as well as patients with anxiety symptoms resulting from physical diseases or mental disorders other than anxiety disorders were excluded from the study. The addiction to psychoactive substances in the last 6 months or cognitive impairment was also the reason for exclusion from the study.

### Study procedure

The HRV examination based on 24-hour Holter ECG recordings is a non-invasive and easy-to-use tool for the assessment of sympathetic-parasympathetic balance within the circulatory system, albeit the interpretation of HRV components and, in particular, its spectral analysis is a subject of ongoing discussion [20].

In the present study, the ambulatory Oxford Medilog MR-63 Holter ECG recorder was used. The HRV analysis was performed with use of the Oxford Medilog Suprima Holter System (Oxford Pol sp. z o.o., Łódź, Poland) (a prospective edition of automatic analysis was applied). The HRV indices were calculated using frequency domain and time domain analysis.

In the HRV spectral analysis, the subject of assessment is both the total power (TP) of the spectrum and its components. According to the current international protocol for HRV method, the following frequency domain parameters are widely used:

– TP (0.00–0.40 Hz) represents the combined tension of both parts of the ANS;
– VLF (0.003–0.04 Hz) partially reflects thermoregulatory mechanisms, fluctuation in the activity of the renin–angiotensin system and the function of peripheral chemoreceptors;
– LF power (0.04–0.15 Hz) is modulated by the sympathetic and partly parasympathetic systems, but is also associated with the activation of arterial baroreceptors;
– HF power (0.15–0.40 Hz) reflects the modulation of vagal tone;
– LF/HF ratio indicates the balance between sympathetic and vagal tone [21].

Time-domain indices of HRV quantify the amount of variability in the measurements of the interbeat interval, which is the time period between successive heartbeats. In the time domain the following parameters were analyzed:

– mean RR (mRR) interval duration between all successive heartbeats,
– SDNN,
– SDNN-i,
– SD of the average NN intervals for each 5 min segment (SDANN),
– rMSSD,
– pNN_50_.

The rMSSD and pNN_50_ are associated with HF power and consequently the parasympathetic activity, whereas SDNN and especially SDNN-i are correlated with VLF and LF power. The SDNN intervals is considered equivalent to the TP total spectral power [21].

The HRV analysis is one of the biomarkers considered to represent the activity of the ANS, especially the parasympathetic nervous system.

### Ethics statement

The studies involving human participants were reviewed and approved by Ethics Committee of Wroclaw Medical University, Wrocław, Poland (Protocol No. KB 58/11). The investigation was conducted in accordance with the World Medical Association's Declaration of Helsinki as revised in 2013. The patients/participants provided their written informed consent to participate in this study after the nature of the procedures had been fully explained.

### Statistical analysis

The obtained values are presented as the M±SD, the median, the min.–max range and interquartile range for continuous variables and numbers with percentages for categorical variables. The variables with non-normal distribution (pNN_50_, TP, VLF, LF, HF) were subjected to logarithmic transformation prior to the statistical analysis in order to obtain normal distribution. The χ^2^ test was used for comparison of dichotomous variables. The ANOVA and Kruskal-Wallis tests were used as appropriate for bivariate analyses. The Shapiro-Wilk test was used in order to study the normality of variables distribution. The pair comparisons were made using the *post hoc* Tukey HSD for ANOVA analyses and Mann-Whitney U test with Bonferroni correction for Kruskal-Wallis *post hoc* analyses. The Statistica software v. 13.3 (StatSoft, Tulsa, OK, USA) was used for the data analysis and the statistical significance was assumed at p < 0.05.

## RESULTS

In the examined groups of academic teachers, 17 patients with PD as a main diagnosis and 21 patients with GAD were enrolled. The personality disorders were the most common comorbidities in the patient groups. Two patients were diagnosed with co-occurring PD and GAD. They were included in the group with a diagnosis of PD, therefore the groups of patients differ as to the presence of PD. None of the patients in the GAD group had panic attacks. Some of the patients in both groups took antidepressants. However, the differences in the frequency of use of medication groups, which were compared using χ^2^ test, were not statistically significant (p = 0.15).

The control group consisted of 40 healthy academic teachers. Persons in the control group did not undergo pharmacological treatment. Characteristics of these groups were depicted in the Table 1.

**Table 1.** Clinical and demographic characteristics of the examined academic teachers, Department of Pathophysiology of Wroclaw Medical University, Wrocław, Poland, 2020–2021

Variable	Participants (N = 78)		
	with PD (N = 17)	with GAD (N = 21)	healthy controls (N = 40)
Age [years]			
Me^a^	37	38	35
min.–max	31–44	32–45	30–43
IQR	34–39	37–40	34–36
M±SD	36.7±4.2	38.5±3.9	35.1±3.4
BMI [kg/m^2^]			
Me^b^	22	22	23
min.–max	17–26	17–25	20–29
IQR	21–24	21–23	22–24
M±SD	22.2±2.6	21.8±2.4	23.05±2.4
Gender [n (%)]			
male	6 (35)	6 (29)	11 (28)
female	11 (65)	15 (71)	29 (72)
The somatic symptoms most commonly reported	– palpitation (100% of patients) – irregular heartbeat (94%) – chest pain (70.5%) – trembling (82%) – sweating (88%) – dizziness (64%) – syncope (23,5%) – increased tiredness (53%) – sleep disorders (70.5%) – restlessness (53%) – breathlessness (59%)	– palpitation (81% of patients) – irregular heartbeat (62%) – chest pain (9.5%) – trembling (24%) – sweating (52%) – dizziness (33%) – syncope (5%) – increased tiredness (52%) – sleep disorders (71%) – restlessness (81%) – breathlessness (9.5%)	no symptom reported
Comorbidities (× number of comorbid disorders)	– GAD (×2) – personality disorders (×5) – somatoform disorder (×1)	– personality disorders (×6) – somatoform disorders (×2)	no comorbidities
Medication^c^ [n of patients]			no medication
SSRI	5	7	
SNRI	2	3	
other	1	1	

GAD – generalized anxiety disorder; PD – panic disorder; SNRI – serotonin norepinephrine reuptake inhibitors; SSRI – selective serotonin reuptake inhibitors.

IQR – inter-quartile range.

Other – benzodiazepines, oxcarbazepine.

Statistical methods: ^a^ Kruskal-Wallis test p = 0.18 (not significant), ^b^ Kruskal-Wallis test p = 0.28 (not significant), ^c^ χ^2^ test p = 0.15 (not significant).

The analysis of the results included in the Table 2 revealed the lowest values of time parameters in the PD group, which were significantly lower than the same parameters in the GAD and control group. The least statistically significant differences were noted between the GAD and control group, where a significant difference concerned only mRR (p = 0.026).

**Table 2.** Comparison of heart rate variability (HRV) time and frequency parameters obtained in panic disorder (PD), generalized anxiety disorder (GAD) and control group during the 24-hour Holter electrocardiogram (ECG) monitoring, Department of Pathophysiology of Wroclaw Medical University, Wrocław, Poland, 2020–2021

HRV parameter	Participants (N = 78)			p		
	with PD (N = 17)	with GAD (N = 21)	healthy controls (N = 40)	PD : GAD	PD : control	GAD : control
Time (M±SD)						
mRR [ms]	652.59±59.37	708.37±64.40	771.34±75.48	**0.049**	**0.002**	**0.026**
SDNN [ms]	98.83±32.19	136.93±21.10	145.07±36.43	**0.016**	**0.003**	0.870
SDNN-i [ms]	35.0±6.79	51.52±8.29	58.73±18.21	**0.017**	**0.011**	0.434
SDANN [ms]	91.23±29.55	126.41±22.53	131.86±35.32	**0.019**	**0.013**	0.855
rMSSD [ms]	15.66±4.61	29.68±7.71	35.13±16.36	**0.018**	**0.002**	0.096
pNN_50_ [%]	0.93±0.63	6.73±3.82	10.63±9.56	**0.013**	**0.009**	0.379
Frequency (M±SD)						
TP [ms^2^]	1580.38±577.08	3013.15±862.62	4251.64±2648.3	0.532	**0.025**	0.356
VLF [ms^2^]	1017.53±421.73	1772.7±515.25	2376.82±1241.43	0.355	**0.005**	0.250
LF [ms^2^]	294.36±97.8	705.01±330.76	1034.41±791.55	**0.012**	**<0.001**	0.605
HF [ms^2^]	79.1±46.29	238.76±148.3	388.85±360.45	**0.006**	**<0.001**	0.651
LF/HF ratio	4.63±2.66	3.4±1.73	3.25±1.47	**0.042**	**0.036**	0.751

HF – high frequency; LF – low frequency; mRR – mean RR interval duration; pNN_50_ – percentage of successive RR intervals that differ by >50 ms; rMSSD – root mean square of successive RR interval differences; SD – standard deviation; SDANN – SD of the average NN intervals for each 5 min; SDNN – SD of NN intervals; SDNN-i – mean of the SD of all the NN intervals for each 5 min; TP – total power; VLF – very low frequency.

Bolded are statistically significant values.

Statistical methods: Shapiro-Wilk test, Mann-Whitney U test, *post hoc* Tukey HSD for 1-way ANOVA analyses.

The comparison of frequency parameters in 24-hour ECG recordings (Table 2) showed the most significant differences between the PD and control group, where the values of TP and its components were significantly lower in the former group. In turn, the significant differences between the GAD and control group were not found. The comparison between the PD and GAD group revealed significant differences in 2 components of the spectrum: LF (p = 0.012) and HF (p = 0.006). However, the LF/HF ratio was significantly higher in the PD group than in the GAD and control group (successively, p = 0.042 and p = 0.036).

The Table 3 contains a comparison of HRV time parameters obtained from day and night recordings in each group. In the PD group, the least statistically significant differences were noted between day and night, which concerned only the SDNN (p = 0.013) and SDANN (p = 0.019) parameters. However, in the GAD group, similarly to the control group, almost all HRV time parameters (except SDNN-i) differed significantly when comparing both recordings.

**Table 3.** Comparison of heart rate variability (HRV) time and frequency parameters obtained in panic disorder (PD), generalized anxiety disorder (GAD) and control group during the 8-hour daytime and 4-hour nighttime Holter electrocardiogram (ECG) monitoring, academic teachers, Department of Pathophysiology of Wroclaw Medical University, Wrocław, Poland, 2020–2021

HRV parameter	Participants (N = 78)						p (daytime : nighttime)		
	with PD (N = 17)		with GAD (N = 21)		healthy controls (N = 40)				
	8-hour daytime	4-hour nighttime	8-hour daytime	4-hour nighttime	8-hour daytime	4-hour nighttime	PD group	GAD group	control group
Time (M±SD)									
mRR [ms]	647.52±60.13	678.21±110.8	655.06±69.19	871.61±101.6	710.95±75.74	926.81±111.4	0.269	**<0.001**	**<0.001**
SDNN [ms]	76.71±18.03	53.20±10.72	92.28±15.95	65.62±9.88	98.19±26.8	87.61±28.78	**0.013**	**0.008**	**0.019**
SDNN-i [ms]	36.79±6.29	28.21±8.61	50.26±12.68	50.08±8.44	54.23±15.97	62.05±25.43	0.056	0.958	0.148
SDANN [ms]	65.86±19.15	41.30±15.37	74.76±17.03	37.85±15.9	79.75±25.97	53.13±23.31	**0.019**	**0.004**	**<0.001**
rMSSD [ms]	15.31±5.32	14.25±6.87	25.95±8.88	36.84±12.41	28.26±10.46	45.22±29.91	0.437	**0.021**	**<0.001**
pNN_50_ [%]	1.06±0.85	0.87±0.61	4.87±3.8	11.99±8.54	7.01±6.68	18.74±19.56	0.257	**0.003**	**<0.001**
Frequency (M±SD)									
TP [ms^2^]	1594.18±512.6	1247.94±707.8	2894.06±1110.3	2771.19±1053.3	3446.83±1986.2	4984.26±4178.6	0.091	0.726	**0.003**
VLF [ms^2^]	1009.35±352.8	826.6±525.87	1636.21±586.26	1568.44±657.37	1923.91±1058.9	2663.01±1721.4	0.255	0.738	**0.001**
LF [ms^2^]	300.78±110.9	286.06±127.43	732.7±484.73	731.93±373.97	920.24±645.53	1153.47±1271.4	0.870	0.995	0.104
HF [ms^2^]	84.37±57.01	65.17±39.28	223.09±205.81	321.44±141.04	259.9±215.4	858.03±1164.4	**0.023**	0.121	**<0.001**
LF/HF ratio	4.42±1.97	7.61±7.94	4.21±2.3	3.23±1.28	4.414±2.041	1.913±1.0	**0.007**	**0.048**	**<0.001**

Abbreviations and explanations as in Table 2.

The Table 3 presents a comparison of HRV frequency parameters obtained from day and night recordings in the study groups. In the PD group, significant differences between daytime and nighttime were noted in the HF component (p = 0.023) and the LF/HF ratio (p = 0.007). In turn, in the GAD group the statistically significant differences concerned only the LF/HF ratio (p = 0.048). It should be noted that the LF/HF ratio was significantly higher in the nighttime recording only in the PD group. A similar comparison in the control group revealed significant differences in the TP value and its components except LF.

## DISCUSSION

Occupational stress among academic teachers has significant effects on their personal health. Prolonged stress can lead to unhealthy lifestyle choices such as physical inactivity which can cause psychogenic cardiovascular conditions [22].

A constantly changing heart rate is an indicator of healthy cardiac regulatory systems that can effectively adapt to sudden environmental and psychological changes. Hence, reduced HRV has been associated with poor health outcomes. The use of HRV in research has become nowadays highly popular due to the ease of HRV recording, as combined with its clinical relevance and significant relationships with cardiovascular diseases and psychophysiological mechanisms of mental disorders and cognitive impairment [23].

It should also be noted that some patients were taking antidepressants that have a different impact on HRV (Table 1). The patients studied were mainly administered selective serotonin reuptake inhibitors (SSRIs). This group of medications is characterized by the limited impact on the ANS and subsequently HRV. The medicines from the serotonin-norepinephrine reuptake inhibitors (SNRI) group with poor or moderate effect on HRV were much less frequently prescribed [24, 25]. Besides, the antidepressants were administered in the examined patients at the lowest therapeutically effective dose level.

The HRV analysis based on 24-hour continuous Holter ECG recordings is the non-invasive method for the assessment of cardiac sympathetic-parasympathetic balance [26]. In the present study, even a preliminary HRV analysis carried out in the PD and GAD group showed the decreased value of mRR parameter which affected directly a decrement of the remaining HRV time parameters as well as heart rate values. The results of the time analysis of the 24-hour Holter ECG recordings (Table 2) demonstrated statistically significant differences between the PD and GAD group in terms of all tested parameters (the examined parameters were significantly lower in the patients with PD as compared with GAD group). The results of the frequency analysis of the 24-hour Holter ECG recordings (Table 2) also showed statistically significant differences between the examined groups in the range of the LF, HF values and LF/HF ratio. The LF and HF values in the PD group were significantly lower than in the GAD group. In turn, the LF/HF ratio was higher in the PD group as reported by other authors [11, 12, 26, 27]. Contrary to the authors' research, Zhang et al. [11] did not find significant association between LF and PD in the LF analysis. Wang et al. [12] reported that GAD patients had significantly lower SDNN than PD patients, albeit the authors of the study performed HRV analysis only with use short-term series (several minutes).

In the authors' study it was noticeable that all parameters of both time and frequency (Table 2) HRV analysis were significantly lower in persons manifesting the symptoms of PD (PD group) as compared to the control group and correlated with symptoms' severity. A decrease in SDNN value below 100 ms, as observed only in these persons, is a relevant predictor of serious cardiac events. However, none of the patients presented with a decrease in SDNN value <50 ms (high risk of cardiac death) [28]. In addition, the decreased SDNN value indicated a predominance of the sympathetic activity as a result of the reduced parasympathetic tone advocated by the decreased HRV parameters of the vagus nerve activity (rMSSD, pNN_50_, HF) [29]. Also, the incidence of cardiac somatic symptoms was higher in the PD group than in the GAD group (Table 1), which may be associated with a decreased protective activity of the vagus nerve in PD patients. In turn, no statistically significant differences were found between the GAD and control group as to the time (except mRR) and frequency parameters on the 24-hour Holter ECG recordings (Table 2 and 3).

According to Paniccia et al. [30], a neurophysiological research on anxiety can be the first step to understanding how physiological flexibility (i.e., HRV) is related to psychological flexibility (i.e., adaptive or maladaptive responses to life events).

The innovative aspect of the present analysis was to find a distinct profile of the HRV with regard to the types of anxiety disorder. Relative to controls, GAD patients had reduced HRV while PD patients displayed the greatest reductions in HRV among the 3 study groups. The characteristic HRV profile was especially clear when comparing the ECG night and daytime recordings (Table 3). It must be stressed once again that the most unfavorable HRV alterations were observed in the persons with PD. These abnormalities comprised the disappearance of day vs. night amplitude of the majority of HRV parameters with a simultaneous significant decrease in their values. In comparison with the control group, the largest decrease was noted in the “night” parameters of the PD group reflecting the vagus nerve activity. These observations were indicative of serious dysfunction of the parasympathetic system resulting in a lack of diurnal rhythm of the sympathetic-parasympathetic regulation. Such considerable abnormalities were not found in the patients with GAD. As to the GAD group, the comparative analysis of the HRV parameters of the day and night recordings showed a decrease in the day vs. night amplitude resulting from attenuation of the parameters related with the sympathetic activity (SDNN, SDANN). However, it was noted only on the night recordings, with a simultaneous increase in the parameters related with the parasympathetic activity (rMSSD, pNN_50_, HF). Such changes were not observed in the persons presenting with symptoms of PD. Contrary to the GAD and control group, instead of increase, a significant decrease in the HRV parameters related to the vagus nerve activity was found at nighttime.

It should be noted that all groups showed significant differences in the LF/HF ratio (Table 3) between the day and night hours, but only in the PD group this difference was unfavorable due to a significantly higher value at night. This indicated the disturbed circadian rhythms within the cardiac sympathetic-parasympathetic regulation resulting from a decrease in nocturnal vagotonia which may subsequently lead to the weakening of antiarrhythmic effect of the vagus nerve. For this reason, the group of patients with panic anxiety were particularly prone to the occurrence of arrhythmic events, including serious ventricular arrhythmias. As compared to traditional biochemical markers of cardiac death or annual routine physical examination, recent studies indicate the higher prognostic value of HRV analysis, whereby its integration with the assessment and monitoring of psychiatric patients during therapy may help to optimize the treatment [31].

Heart rate variability is a useful clinical marker of psychopathology, which represents cardiac sympathetic-parasympathetic balance, reflecting an adaptive response to external and internal changes. A decrease in HRV as observed in persons with anxiety disorders is a sign of heart autonomic dysregulation. Although the specific pathophysiological mechanisms underlying the associations between psychiatric conditions and HRV remain poorly understood, the majority of studies observed that reduced vagal activity could be a diagnostic biomarker of psychiatric disorders [22, 32, 33].

In the academic teachers diagnosed with anxiety disorders, the components of cognitive behavioral therapy depend on the type of disorder and the therapist's style. In the Wrocław Center for Mental Health, the preferred model is based upon adaptation to external conditions with the simultaneous minimization of internal costs of such adaptation. It allows for the optimal psychophysical balance between the patient and the environment. The main emphasis is put on the intensive intra- and interpersonal training and analytically-oriented group therapy. The intrapersonal training began with psychoeducation, self-observation and autogenic training. Patients are taught to correct the usually exaggerated importance of anxiety and the somatic symptoms associated with it during psychoeducation. Self-observation allows to create the own objective model of reaction to anxiety. However, autogenic training allows to control these reactions. It includes progressive muscle relaxation exercises and breathing relaxation techniques. As a result of the training, the autonomic system balance is restored, but the psychological aspect of the autogenic training is also important. The intrapersonal training has a positive effect on proper relationships with other people, and the subsequent stages of behavioral and cognitive therapy, i.e., interpersonal training and group therapy serve to consolidate these relationships [34].

### Study limitations

The study groups were relatively small, because the patients came from one Center for Mental Health and were limited to one professional group. Moreover, the imposed exclusion criteria were very restrictive.

The advantage of the patients' selection procedure is a detailed psychiatric diagnosis, which allows for the distinction between patients with PD and those with GAD. However, the intensity of disorders was not estimated (only study entry criteria requirements were used).

The specific circumstances of the study are another limitation, i.e., the patients participated in the group therapy, which affected their emotions in the way that is difficult to define. The patients with PD and GAD were in the same situation, which authenticated the comparisons between them. The control group came from population, which did not undergo the group therapy.

The lack of control over potential confounding factors like physical activity and sleep patterns.

## CONCLUSIONS

To summarize, the data obtained in this study confirm that the circadian rhythms of HRV as assessed with the Holter ECG monitoring are considerably flattened in patients with anxiety disorders. In the PD group, the largest decrease was noted in the “night” parameters reflecting the vagus nerve activity, which resulted in a lack of diurnal rhythm of the sympathetic-parasympathetic regulation. Such considerable abnormalities were not found in the persons with GAD, where a small increase in nocturnal vagotonia was observed. The increase was smaller than in the control group, albeit it was significantly bigger as compared with the PD group. In addition, the significant differences in the day vs. night LF/HF ratio were found in all groups, but only in the PD group the difference was unfavorably big. The assessment of the impact of anxiety disorders on the circadian HRV requires differentiating between PD and GAD because of the considerable differences related to the nature of the particular disorders.

The authors' results suggest that reduction in HRV could be an objective biological marker to distinguish between PD and GAD patients, based on the psychophysiological correlates such as HF component. The considerably flattened circadian rhythm of HRV profile should be considered as a biomarker of cardiac autonomic inflexibility in different types of anxiety disorder. Heart rate variability enables not only a prognostic assessment related to the current health condition, but also allows for control of the basic components of heart rate regulation both during periods of psychotherapy and the exacerbation of anxiety disorders.

In conclusion, anxiety disorder in the academic teachers is a serious problem that concerns both physical and mental health. Hence, this condition requires a comprehensive approach, and cognitive behavioral therapy is an important element of it.

Ensuring adequate support that includes access to medical and psychological care and creating a favorable workplace environment can help to alleviate symptoms and improve on the quality of their professional and private life [1].

## References

[1] Agyapong B, Obuobi-Donkor G, Burback L, Wei Y. Stress, burnout, anxiety and depression among teachers: A scoping review. Int J Environ Res Public Health. 2022;19(17):10706. 10.3390/ijerph191710706. 36078422 PMC9518388

[2] Järvelin-Pasanen S, Sinikallio S, Tarvainen MP. Heart rate variability and occupational stress-systematic review. Ind Health. 2018;56(6):500–511. 10.2486/indhealth.2017-0190. 29910218 PMC6258751

[3] Clark DA, Beck AT. Cognitive theory and therapy of anxiety and depression: convergence with neurobiological findings. Trends Cogn Sci. 2010;14(9):418–24. 10.1016/j.tics.2010.06.007. 20655801

[4] Siegel EH, Sands MK, Van den Noortgate W, Condon P, Chang Y, Dy J, et al. Emotion fingerprints or emotion populations? A meta-analytic investigation of autonomic features of emotion categories. Psychol Bull. 2018;144(4):343–393. 10.1037/bul0000128. 29389177 PMC5876074

[5] Dawson DK. Acute stress-induced (takotsubo) cardiomyopathy. Heart. 2018;104:96–102. 10.1136/heartjnl-2017-311579. 28824005

[6] Hering D, Lachowska K, Schlaich M. Role of the sympathetic nervous system in stress-mediated cardiovascular disease. Curr Hypertens Rep. 2015;17:80. 10.1007/s11906-015-0594-5. 26318888

[7] Esler M. Mental stress and human cardiovascular disease. Neurosci Biobehav Rev. 2017;74:269–76. 10.1016/j.neubiorev.2016.10.011. 27751732

[8] Manolis AA, Manolis TA, Apostolopoulos EJ, Apostolaki NE, Melita H, Manolis AS. The role of the autonomic nervous system in cardiac arrhythmias: The neuro-cardiac axis, more foe than friend? Trends Cardiovasc Med. 2021;31:290–302. 10.1016/j.tcm.2020.04.011. 32434043

[9] Cheng YC, Su MI, Liu CW, Huang YC, Huang WL. Heart rate variability in patients with anxiety disorders: A systematic review and meta-analysis. Psychiatry Clin Neurosci. 2022;76:292–302. 10.1111/pcn.13356. 35340102

[10] Ramesh A, Nayak T, Beestrum M, Quer G, Pandit JA. Heart rate variability in psychiatric disorders: a systematic review. Neuropsychiatr Dis Treat. 2023;19:2217–39. 10.2147/NDT.S429592. 37881808 PMC10596135

[11] Zhang Y, Zhou B, Qiu J, Zhang L, Zou Z. Heart rate variability changes in patients with panic disorder. J Affect Disord. 2020; 267:297–306. 10.1016/j.jad.2020.01.132. 32217230

[12] Wang Z, Luo Y, Zhang Y, Chen L, Zou Y, Xiao J, et al. Heart rate variability in generalized anxiety disorder, major depressive disorder and panic disorder: A network meta-analysis and systematic review. J Affect Disord. 2023;330:259–66. 10.1016/j.jad.2023.03.018. 36914118

[13] McCraty R, Atkinson M, Tomasino D, Stuppy WP. Analysis of twenty-four hour heart rate variability in patients with panic disorder. Biol Psychol. 2001;56:131–50. 10.1016/s0301-0511(01)00074-6. 11334700

[14] Gündüz N, Akpınar Aslan E, Eren F, Sodan Turan H, Öztürk M, Tural Ü. Analysis of 24-hour heart rate variability among panic disorder patients without previous drug treatment and comorbid disorders. Turk Psikiyatri Derg. 2019;30:236–44. 32594484

[15] Sloan EP, Natarajan M, Baker B, Dorian P, Mironov D, Barr A, et al. Nocturnal and daytime panic attacks – comparison of sleep architecture, heart rate variability, and response to sodium lactate challenge. Biol Psychiatry. 1999;45:1313–20. 10.1016/s0006-3223(98)00158-9. 10349038

[16] Karasek R, Collins S, Clays E, Bortkiewicz A, Ferrario M. Description of a large-scale study design to assess work-stress-disease associations for cardiovascular disease. Int J Occup Med Environ Health. 2010;23(3):293–312. 10.2478/v10001-010-0035-2. 21306975

[17] Schützwohl M, Kallert T, Jurjanz L. Using the schedules for clinical assessment in neuropsychiatry (SCAN 2.1) as a diagnostic interview providing dimensional measures: cross-national findings on the psychometric properties of psychopathology scales, Eur Psychiatry. 2007;22:229–38. 10.1016/j.eurpsy.2006.10.005. 17188845

[18] Małyszczak K, Rymaszewska J, Hadryś T, Adamowski T, Kiejna A. [Comparison between a SCAN diagnosis and a clinical diagnosis]. Psychiatr Pol. 2002;36:377–80. Polish. 12647462

[19] Adamowski T, Kiejna A, Hadryś T. [Study of compatibility of psychiatric diagnoses with ICD-10 diagnostic criteria using the SCAN questionnaire]. Psychiatr Pol. 2006;40:761–73. Polish. 17068948

[20] Li K, Rüdiger H, Ziemssen T. Spectral analysis of heart rate variability: time window matters. Front Neurol. 2019:10:545. 10.3389/fneur.2019.00545. 31191437 PMC6548839

[21] Task Force of the European Society of Cardiology and the North American Society of Pacing and Electrophisiology. Heart rate variability: standards of measurement, physiological interpretation and clinical use. Circulation. 1996;93:1043–65. 10.1161/01.CIR.93.5.1043. 8598068

[22] Balachandran K, Maniyara K, Palle E, Kodali PB. Stress and hypertension among university teachers: A cross-sectional survey from Northern Kerala. Indian J Occup Environ Med. 2025;29:15–20. 10.4103/ijoem.ijoem_62_24. 40275899 PMC12017665

[23] Pham T, Lau ZJ, Chen SHA, Makowski D. Heart rate variability in psychology: a review of HRV indices and an analysis tutorial. Sensors (Basel). 2021;21:3998. 10.3390/s21123998. 34207927 PMC8230044

[24] Alvares GA, Quintana DS, Hickie IB, Guastella AJ. Autonomic nervous system dysfunction in psychiatric disorders and the impact of psychotropic medications: a systematic review and meta-analysis. J Psychiatry Neurosci. 2016;41:89–104. 10.1503/jpn.140217. 26447819 PMC4764485

[25] Iwamoto Y, Kawanishi C, Kishida I, Furuno T, Fujibayashi M, Ishii C, et al. Dose-dependent effect of antipsychotic drugs on autonomic nervous system activity in schizophrenia. BMC Psychiatry. 2012;12:199. 10.1186/1471-244X-12-199. 23151241 PMC3534356

[26] Durdu GŞ, Kayikcioğlu M, Pirildar Ş, Köse T. Evaluation of heart rate variability in drug free panic disorder patients. Noro Psikiyatr Ars. 2018;55:364–69. 10.5152/npa.2017.19429. 30622395 PMC6300828

[27] Mumm JLM, Pyrkosch L, Plag J, Nagel P, Petzold MB, Bischoff S, et al. Heart rate variability in patients with agoraphobia with or without panic disorder remains stable during CBT but increases following in-vivo exposure. J Anxiety Disord. 2019;64:16–23. 10.1016/j.janxdis.2019.03.001. 30875662

[28] Shaffer F, Ginsberg JP. An overview of heart rate variability metrics and norms. Front Public Health. 2017;5:258. 10.3389/fpubh.2017.00258. 29034226 PMC5624990

[29] Koch C, Wilhelm M, Salzmann S, Rief W, Euteneuer F. A meta-analysis of heart rate variability in major depression. Psychol Med. 2019;49:1948–57. 10.1017/S0033291719001351. 31239003

[30] Paniccia M, Paniccia D, Thomas S, Taha T, Reed N. Clinical and non-clinical depression and anxiety in young people: A scoping review on heart rate variability. Auton Neurosci. 2017;208:1–14. 10.1016/j.autneu.2017.08.008. 28870754

[31] Pernaje Seetharam S, Shankar MV, Udupa KAR, Reddy N. Prognostic value of heart rate variability in acute coronary syndrome. J Basic Clin Physiol Pharmacol. 2022;34:337–47. 10.1515/jbcpp-2022-0134. 36194293

[32] Chalmers JA, Quintana DS, Abbott MJ, Kemp AH. Anxiety disorders are associated with reduced heart rate variability: a meta-analysis. Front Psychiatry. 2014;5:80. 10.3389/fpsyt.2014.00080. 25071612 PMC4092363

[33] Hartmann R, Schmidt FM, Sander C, Hegerl U. Heart rate variability as indicator of clinical state in depression. Front Psychiatry. 2019;9:735. 10.3389/fpsyt.2018.00735. 30705641 PMC6344433

[34] Chładzińska-Kiejna S, Małyszczak K. Terapia behawioralna i poznawcza – podstawowe założenia, cele i techniki terapeutyczne. In: Kiejna A, Małyszczak K, editors. Psychiatria. Podręcznik akademicki. Wrocław: Akademia Medyczna im. Piastów Śląskich; 2011. p. 303–19.

